# Interconnectedness Is Associated with a Greater Sense of Civic Duty and Collective Action Participation through Transcendental Awareness and Compassion during COVID-19

**DOI:** 10.3390/ijerph19127261

**Published:** 2022-06-14

**Authors:** Winnie W. S. Mak, Sin Man Ng, Emily W. S. Tsoi, Ben C. L. Yu

**Affiliations:** Department of Psychology, The Chinese University of Hong Kong, Shatin, NT, Hong Kong; mandynsm@gmail.com (S.M.N.); tsoi.emily@gmail.com (E.W.S.T.); clyuben@gmail.com (B.C.L.Y.)

**Keywords:** COVID-19, pandemic, interconnectedness, self-transcendence, compassion, civic duty, collective action

## Abstract

The COVID-19 pandemic has a unprecedented impact on the way individuals make sense of the interconnected nature of themselves in relation to the world. This study investigated the mediating role of transcendental awareness and compassion in the association of interconnectedness with a sense of civic duty and collective action participation during COVID-19 using a longitudinal design. A total of 336 young adult participants were recruited at baseline and were asked to complete measures of interconnectedness, transcendental awareness, compassion, civic duty, and collective action participation at three time points over a 6-month period. Path analysis was used to test the hypothesized mediation model. The results showed that compassion fully mediated the positive association between interconnectedness and collective action participation and partially mediated the positive association between interconnectedness and civic duty. Transcendental awareness also partially mediated the positive association between interconnectedness and civic duty but not collective action participation. This study highlighted the potential of interconnectedness in promoting civic duty and engagement in collective action through transcendental awareness and compassion during the COVID-19 pandemic.

## 1. Background

The coronavirus (COVID-19) pandemic that began in 2019 is said to cause more “mass trauma” than World War II [1], marking it one of most significant events in modern history. Undoubtedly, its impact has reached a global scale that is unparalleled. Governments, institutions, communities, and individuals across the world are impacted in different ways, at both individual and collective levels. At the individual level, apart from the interrelated health, mental health, economic, sociopolitical ramifications, the outbreak puts substantial pressures on individuals and members of the communities to challenge their priorities and change their behaviors for individual and collective good. At the collective level, COVID-19 illuminates the complex interdependent relationships of governments, economies, globalization, civic duty, and public health.

Disasters, whether natural or human-caused, or other incidents of mass trauma such as a worldwide pandemic can cause a myriad of direct (e.g., economic) and indirect (e.g., psychological) consequences on affected populations [2]. COVID-19 wreaks havoc on the global economy by affecting production, disrupting supply chains and markets, decreasing trading, and bringing down the global GDP [3]. The COVID-19 pandemic also has tremendous short-term and long-term ramifications on mental health and health [4]. Despite the deleterious impacts brought about by this global crisis, the collective experience also prompts individuals and countries to contemplate on their mutual roles and possible changes that the collective can bring upon to the world. COVID-19 accelerates global leaders and citizens to reflect on the interdependent nature of all beings along the biopsychosocial, economic, political, and environmental spheres [5,6]. Under the threat of a “common enemy”, self-serving behaviors may be detrimental to the public and have been demonstrated to be associated with loneliness and depressive symptoms [7,8]. Governments across the globe also put forth various measures in containing the spread of the virus [9].

In fact, emerging evidence suggested that the pandemic brought about a sense of community among fellow citizens [10,11]. To date, a handful of commentaries urges us to reflect on and learn from the observed difficulties (e.g., unequal distribution of medical and financial resources globally) during the pandemic that have been contributed by entrenched structural problems, including health inequities, climate injustice, and unequal global economic order and to build our future collectively in the post-pandemic world [12,13]. Similarly, articles about the interdependent nature of institutions, global economies, and health systems have been published, calling for collective action for positive changes to safeguard the world from future crises [14,15]. However, relatively little empirical work has been done to understand the ways in which this pandemic has prompted individuals to rethink our interdependent role in the world, which may nurture reflections that transcend oneself, cultivate compassion, and promote one’s sense of civic duty and collective action participation.

### 1.1. From Me to We: The Impact of Interconnectedness during the Pandemic

COVID-19 has shed light on the interconnected nature of all phenomena, raising individual and societal awareness on how our behaviors can affect each other. This is consistent with ideas espoused by existential positive psychology, whereby individuals tend to re-negotiate their assumptions about the world when confronted by adversities that threaten our understanding of the self and our place in the world [16,17]. Indeed, one of the prerequisites for altruistic actions is when individuals become sensitive to the needs and welfare of others and think in collective rather than personal terms [18,19]. One of the upshots of COVID-19 is the realization that we are all interconnected with each other in the world, and this realization may propel us to shift our considerations to be inclusive of others rather than solely focusing on ourselves in our approach to civic affairs and social issues as none of us are isolated in the world and our actions have consequences to each other, especially in improving the social conditions of the society.

Adopted from Buddhist philosophy, interconnectedness has been operationalized as “an awareness that the existence of all phenomena in the world is the result of the fulfilment of different causes and conditions, in which no entity can substantiate independently without relying on other factors” [20]. Cross-sectionally, interconnectedness was found to be positively associated with mental well-being, social connectedness, compassion, self-transcendence, and beliefs concerning social justice and equality [20]. Experimentally, the induction of interconnectedness led to compassion, which in turn predicted intention to advocate for the rights of people with mental illness [21]. Another experimental study observed that cultivation of interconnectedness can confer on people a sense of responsibility over recovery of people with mental illness, which in turn predicted the intention to advocate for the rights of people with mental illness at a later time point [22]. These studies altogether suggested that being aware of the interconnected nature of all matters may bring about benefits both intrapersonally and interpersonally and it may be related to being self-transcendent and compassionate.

Transcendental awareness is having the realization and appreciation of the intricate nature of oneself in relation to the world at multiple perspectives (economics, public health, culture, environment, etc.) during the COVID-19 pandemic. It is a perception and understanding of the world that is beyond one’s self-interest, with a sense of connectedness and relationships with other individuals and ecology in the world [23]. Having an awareness of this interconnectedness in the world, individuals may develop transcendental awareness and compassion that fuel their intention to serve the collective good. In particular, individuals may increase in their sense of civic duty, which is a sense or cognition that an action is beneficial to others, thus obligatory to the self to act even though it may yield a cost to the individual participating in the action [24]. They may also participate in collective action that can be understood as a means for individuals to advance group interests and contribute socially to the public good [25,26]. It includes any action that aims to improve the status, welfare, and state of affairs of an entire group or population, rather than that of one or a few individuals. The capacity to care for other people in the society and concern for social causes that may benefit fellow citizens in the society had been demonstrated to correlate with finding meaning in adversity and mental well-being, both pre-pandemic and during the pandemic [7,27,28]. In fact, a qualitative study posited that the sense of self-transcendence that has emerged amidst the pandemic could be conceptualized as “the expression of and the creation of a sense of relatedness to others” [29]. Another cross-sectional study also demonstrated self-transcendence to be associated with prosocial intentions and behaviors (e.g., willingness to do grocery shopping for neighbors who are in need and telework for people in developing countries during the pandemic) and to fully mediate the relationship between social dominance orientation (SDO) and prosociality [30]. SDO refers to the extent to which an individual supports social hierarchy and wants one’s in-group to dominate and be superior to outgroups [31]; prosocality is defined as voluntary actions that one takes to help, assist, or comfort other people [32].

Compassion is an approach-oriented emotion that entails a feeling of care toward those who are suffering and a motivation to relieve the suffering [33,34]. According to the Halifax Model of Compassion [35], compassion is cultivated through an emergent process within a sociopolitical context and in essence, this emotional sentiment is transcendental, concerning all people other than close others and are conducive to the promotion of social justice and stigma reduction [21,36,37]. According to Lomas [38], “compassion allows one to experience self-transcendence and enter into a more ‘intersubjective’ mode of identity”. Compassion was found to be an important antecedent to prosocial behaviors and collective action [39,40,41,42].

### 1.2. The Present Study

Based on the literature reviewed, the present study aimed to investigate the relationship between interconnectedness, transcendental awareness, compassion, civic duty, and collective action participation in the context of the COVID-19 pandemic using a 6-month longitudinal design across three time points. We hypothesized that interconnectedness at baseline is associated with greater transcendental awareness at Time 2 (3rd month) and greater compassion at Time 2 (3rd month), which in turn is associated with greater civic duty and collective action participation at Time 3 (6th month).

## 2. Methods

### 2.1. Participants and Procedure

The present study is part of a larger research study named Mindful Flourishing that aimed to promote mindfulness and flourishing among young adults in Hong Kong through a mindfulness ambassador program and a mindfulness-related mobile application [43]. Upon obtaining ethics approval from the Survey and Behavioral Research Ethics Committee of the Chinese University of Hong Kong, research participant recruitment was sent through mass email of the university and posts on social media. Participants were invited to read a detailed consent form online through Qualtrics, an online questionnaire software, and provided their consent to the research terms by clicking on the agree button before completing a set of online questionnaires, at the beginning of the study (Time 1), 3-month (Time 2), 6-month (Time 3), and 9-month of the study (Time 4) between March 2020 to November 2020. Specifically, the exact dates for each wave of data collection were 3–31 March (Time 1), 19 May–10 June (Time 2), 20 July–10 August (Time 3), and 25 September–12 November 2020 (Time 4). Upon completion of the questionnaires, participants received coupons valuing HK$160 in total. For the first two time points, they received a coupon of HK$50 for each set of questionnaires and two coupons of HK$30 were reimbursed if participants completed the remaining two sets of questionnaires (US$1 ≈ HK$7.8).

A total of 336 participants gave their informed consent after they were given written instructions and explanation of the study online. The response rates of these 336 participants were 90% (Time 2), 82% (Time 3), and 80% (Time 4). Since 31 participants only completed the baseline questionnaires but not any of the subsequent time points, they were excluded from analyses, resulting in a final sample size of 305. The present study used the first three waves of data to test the mediation model. The age range of the participants was from 18 to 38 years old (M = 21.33, SD = 2.96); 258 (85.7%) of them were female; 237 (78.0%) of them indicated their education level as undergraduate and 22 (7.4%) of them as postgraduate; 212 (70.4%) of them had no religion while 9 (3.0%) of them were Buddhist and 77(25.7%) were Christian or Catholic; 165 (54.8%) of them had never tried any mindfulness exercises before they participated in the study.

### 2.2. Measures

Participants were asked to complete the following questionnaires at all time points except that the measure of transcendental awareness was administered only from Time 2–4.

#### 2.2.1. Interconnectedness

The 12-item Interconnectedness Scale [20] that was originally developed in Chinese was used to assess levels of interconnectedness. Participants rated their extent of agreement on views about the interconnected nature of the world on a 6-point scale (1 = totally disagree, 6 = totally agree). Four items assessed emotional response to interconnectedness (ERI), another four items measured appreciation of interconnectedness on self-development (AIS), with the remaining four items assessing interconnectedness in social relations (ISR). Composite score of the scale was used in the present study, which was computed by averaging the scores of the 12 items with higher scores showing higher levels of interconnectedness. Sample items are “Since everything in this world is affecting one another, I actively pay attention to every happening in the world (ISR).”, “I do not blame myself when things go wrong because the result is contributed by many factors (ERI).”, and “I understand that it takes numerous factors working together to succeed, so I cannot fully control the result (AIS).” The scale was positively correlated with mental well-being, nonattachment, as well as universalism and active engaged citizenship [20]. It has been found to be reliable among samples of students in Hong Kong (Cronbach’s α = 0.78–0.87 [20]). The test-retest reliability with a 4-week interval was 0.72 [20].

#### 2.2.2. Transcendental Awareness during COVID-19

Eight items in Chinese were constructed by the authors to examine respondents’ transcendental awareness over the past three months during COVID-19. The items were assessed on a 6-point scale on degree of agreement (1 = totally disagree, 6 = totally agree). Composite score was obtained by averaging the scores of all eight items. Higher scores indicated greater agreement on having thoughts about the interconnected nature between people and society across the globe during COVID-19. The items are developed based on the interconnected conditions raised in previous commentaries about the pandemic [12,13,14,15]. Together with the instruction “In the past three months, the COVID-19 pandemic has prompted me to ……”, the 8 items are “think about the meaning of life”, “think about the connection between people”, “think about the situations of people from different social classes”, “think about the relationship between personal hygiene and public health”, “think about cultural differences around the world”, “think about the economic systems in the society”, “think about the governance effectiveness of the government”, and “think about the global environmental issues”.

#### 2.2.3. Compassion

The 5-item Santa Clara Brief Compassion Scale (SCBCS) [44] was used to measure levels of compassion. The scale was originally derived from the Compassionate Love Scale [45] that measures compassionate love toward others. It was translated into Chinese by the research team using the forward-back translation approach. Participants rated the extent of how true the statements were for them on a 7-point scale (1 = not at all true of me, 7 = very true of me). Composite score was obtained by averaging the scores of all five items with higher scores indicating higher levels of compassion. Sample items are “When I hear about someone (a stranger) going through a difficult time, I feel a great deal of compassion for him or her” and “I often have tender feelings toward people (strangers) when they seem to be in need”. The scale was positively related to personal accomplishment [46], as well as compassionate love and empathy [44]. The internal consistency of the scale has been demonstrated in samples of students from the US (Cronbach’s α = 0.89–0.90) [44,46,47].

#### 2.2.4. Civic Duty

The 12-item civic duty subscale from the Active and Engaged Citizenship Scale [48] was used to measure the sense of civic duty. The Chinese version of the subscale was back-translated by the research team. Participants rated their extent of agreement on the first five statements on a 5-point scale (0 = not important, 4 = extremely important) and the remaining seven items on another 5-point scale (0 = strongly disagree, 4 = strongly agree). Two of the items were reverse scored. Composite score was obtained by averaging all the scores of 12 items with higher scores indicating a greater sense of civic duty. Sample items are “helping to make the world a better place to live in” and “It is important to me to contribute to my community and society”. The scale was positively correlated with parental warmth and political discussion [49]. It has been found to be reliable in samples of students from the US (Cronbach’s α = 0.86–0.87 [48]).

#### 2.2.5. Collective Action Participation

The Collective Action Scale was adapted from the scale used among collective action for sexual minorities in Hong Kong [50]. The 14-item Chinese version of Collective Action Scale examined the frequency of participation in collective action among people in general. Participants assessed their frequency of actual participation in collective action on a 5-point scale (1 = never, 5 = frequently). Composite score was obtained by averaging the scores of all 14 items. Higher scores indicated more frequent actual participation in collective action. Sample items are “Discuss social issues with family and/or friends to raise their awareness of the rights of members in the community” and “Participate in petitions (including online petitions) to advocate for the rights of members in the community”. The scale was found to be related to more frequent experience of discrimination among a sample of lesbians, gays, bisexual, and transgender individuals (LGBT) in Hong Kong [50]. The internal consistency of the scale has been demonstrated in the LGBT sample from Hong Kong (Cronbach’s α = 0.71−0.90 [50]).

### 2.3. Data Analyses

Analysis on Pearson correlation among the study variables was conducted using SPSS (Version 25). Path analysis was used to test the hypothesized mediation model using Mplus Version 8.7 [51]. Interconnectedness at Time 1 was structured as an independent variable; transcendental awareness at Time 2 and compassion at Time 2 were entered as mediators; and civic duty and collective action participation at time 3 were treated as dependent variables.

Bootstrapping technique was used to test the hypothesized indirect effects of the mediation model [52]. About 5000 bootstrap samples were derived from the original data (N = 305) to generate bias-corrected 95% confidence intervals (BC 95% CI) in order to obtain accurate mediating effects assessment [53]. If the indirect effects were significant at *p* < 0.05 and their BC 95% CI did not include zero, the hypothesized mediating effects were supported [52]. To evaluate data-model fit, the chi-square statistics (χ2), Comparative Fit Index (CFI), Tucker-Lewis Index (TLI), root mean square error of approximation (RMSEA), and standardized root mean square residual (SRMR) were used [54]. If both CFI and TLI > 0.90, RMSEA and SRMR < 0.08, the hypothesized mediation model would be accepted [54,55]. Missing data were addressed by full maximum likelihood estimation.

## 3. Results

### 3.1. Descriptive Statistics

The bivariate associations between the mindfulness practice experience, interconnectedness, transcendental awareness, compassion, civic duty, and collective action participation were investigated by Pearson correlation. The results showed that mindfulness practice experience was significantly associated with the four studied variables, while most of the studied variables were significantly correlated with one another. Given the significant association of mindfulness practice experience with other variables, past studies have shown that mindfulness is associated with interconnectedness, compassion, and self-transcendence [20]; mindfulness practice experience was controlled in the mediation analysis. Means, standard deviations, zero-order correlations, and internal consistency of the study variables are shown in Table 1.

### 3.2. Mediation Model

**Figure 1:**
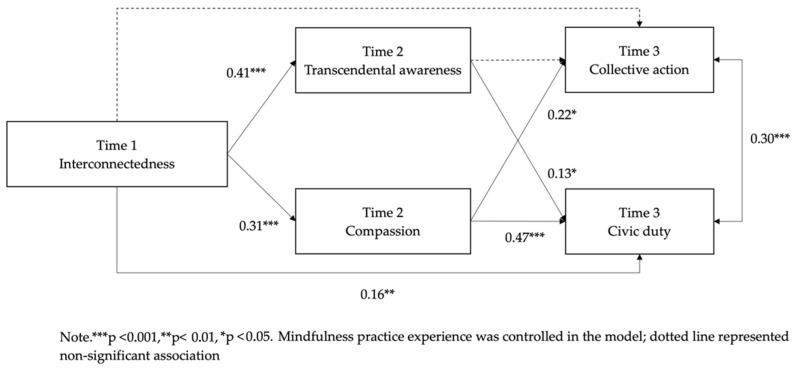
Standardized estimates of the hypothesized model.

Result suggested satisfactory goodness-of-fit for the mediation model: χ^2^ (1) = 2.60, *p* = 0.11; CFI = 0.99; TLI = 0.91; RMSEA = 0.07; SRMR = 0.02 (Figure 1). The associations of interconnectedness at Time 1 with transcendental awareness at Time 2 (*β* = 0.41, *p* < 0.001, 95% CI 0.28 to 0.52) and with compassion at Time 2 (*β* = 0.31, *p* < 0.001, 95% CI 0.19 to 0.41) were both significant (see Figure 1). After controlling for interconnectedness at Time 1 and transcendental awareness at Time 2, the association of compassion at Time 2 with civic duty (*β* = 0.47, *p* < 0.001, 95% CI 0.36 to 0.57) and collective action participation at Time 3 (*β* = 0.22, *p* < 0.001, 95% CI 0.10 to 0.32) remained significant. After controlling for interconnectedness at Time 1 and compassion at Time 2, the association of transcendental awareness at Time 2 remained significantly associated only with civic duty at Time 3 (*β* = 0.13, *p* = 0.02, 95% CI 0.03 to 0.25) but not collective action participation at Time 3 (*β* = 0.06, *p* = 0.39, 95% CI −0.08 to 0.20). The direct effect of interconnectedness at Time 1 on civic duty at Time 3 (*β* = 0.16, *p* = 0.01, 95% CI 0.04 to 0.28), but not on collective action participation at Time 3 (*β* = 0.001, *p* = 0.99, 95% CI −0.12 to 0.11), was significant. 

The specific indirect effects of interconnectedness at Time 1 on both civic duty (*β* = 0.14, *p* < 0.001, 95% CI 0.09 to 0.21) and collective action participation at Time 3 (*β* = 0.07, *p* = 0.003, 95% CI 0.03 to 0.12) through compassion at Time 2 were all significant. Through transcendental awareness, the indirect effect of interconnectedness at Time 1 on civic duty at Time 3 (*β* = 0.05, *p* = 0.04, 95% CI 0.01 to 0.11), but not on collective action participation at Time 3 (*β* = 0.03, *p* = 0.41, 95% CI −0.03 to 0.09) was significant. Altogether, the results implied that compassion and transcendental awareness partially mediated the association between interconnectedness and civic duty. Furthermore, compassion fully mediated the association between interconnectedness and collective action participation.

## 4. Discussion

### 4.1. Major Findings

The present study investigated the mediating roles of transcendental awareness during the pandemic and compassion in the relationships of interconnectedness with civic duty and collective action participation on a sample of young adults longitudinally over a 6-month period. Our findings supported the potential of transcendental awareness and compassion as mechanisms underlying the effect of interconnectedness on a sense of civic duty and compassion on the effect of interconnectedness on collective action participation for young adults in Hong Kong. The mediation model was generally supported and several major findings are worth highlighting. First, this study supported the positive relationship between interconnectedness and civic duty. This is consistent with the findings from Yu et al. [20] that found interconnectedness to be associated positively with beliefs in social justice and prosocial behaviors. Second, even though a direct relationship between interconnectedness and collective action participation cannot be found, our results highlighted the significant role compassion plays in mediating individuals’ levels of interconnectedness with both civic duty and collective action participation. Collective action, by definition, is traditionally conceptualized as an intergroup behavior [25,56], and compassion alludes to sentiments that are directed to all human beings regardless of group status [45]. Our findings are in line with recent research on compassion where it was found to reduce intergroup conflict and stigma in minority groups [57,58]. Moreover, the findings supported the hypothesized relationships that people who are more aware of the interconnected nature of all matters are more aware of the interconnectedness of people and systems during the pandemic and are more compassionate. Between the mechanisms of transcendental awareness and compassion, compassion had a more potent effect on both civic duty and collective action participation. Whereas transcendental awareness is mainly a cognitive acknowledgment on the interconnected relationships between individuals and systems during the pandemic, compassion encompasses cognitive, affective, motivational, and behavioral dimensions that may be able to arouse one’s sense of civic duty and to partake in collective action for collective well-being through multiple psychological pathways [59]. Future studies may unpack these compassion pathways to examine which one has the strongest effect in promoting civic duty and prosocial behavioral changes.

The results suggested that these mechanisms warrant further investigation to examine how we can more effectively motivate one to contribute to the common good in the society. This might be achieved by examining the distinct contributions between interconnectedness, transcendental awareness, and compassion on prosociality and collective action using experimental induction or specific interventions [21]. In addition, contextual factors could also be captured to understand how or whether changing life circumstances or goals may affect a person’s sense of interconnectedness, compassion, and thus their willingness to extend help for others. In sum, further research should be carried out to delineate the theoretical relationships between these constructs and testing them empirically.

### 4.2. Implications

The present study carries several practical implications. Past research has shown contemplative practices such as mindfulness, loving-kindness, or compassion meditation to increase positive emotions, social connectedness, decrease mental distress and physical stress as indicated by biomarkers [60,61,62]. Therefore, interventions that cultivate mindful awareness, the arising of interconnectedness, and compassion can be designed to potentially enhance individuals’ sense of connections and responsibility to alleviate suffering, which can support individuals to serve the common good [21,22,63]. On a larger scale, public health initiatives can also help to facilitate recovery by framing COVID-19 as a collective and shared experience through an empathetic, interconnected, and supportive lens to promote psychosocial growth in the public amidst threats from the pandemic.

### 4.3. Limitations 

The results should be interpreted with its limitations in mind. First, transcendental awareness during COVID-19 is measured using self-constructed items that were not previously validated. Second, this study was conducted with a group of young adults who are predominantly female, thus, generalizability of the results should be made with caution. The study was also correlational in nature and could at best illuminate on temporal relationships; causality cannot be drawn. Notwithstanding these limitations, our study provided valuable insights into understanding how interconnectedness may be related to being compassionate and having transcendental awareness that may arise during the pandemic and how they may be harnessed to improve a sense of civic duty and encourage collective action participation.

## 5. Conclusions

At the time of writing, the world is still adapting to the COVID-19 pandemic. With a new virus variants spawning, the pandemic continues to pose uncertainties and expose vulnerabilities in our communities [64,65]. Our results point to the possibility of shifting from being self-focused to be more collective-oriented to serve the public; it is hoped that this study will stimulate further research in this area and promote an awareness of the interconnectedness nature between all beings in the world in spite of geographic, social, and health spectra, to bring about positive transformation for collective good.

## Figures and Tables

**Table 1:** Descriptive statistics and correlations of the study variables.

Variables	1	2	3	4	5
1. Interconnectedness (Time 1)	-				
2. Transcendental awareness (Time 2)	0.42 ***	-			
3. Compassion (Time 2)	0.32 ***	0.24 **	-		
4. Civic duty (Time 3)	0.38 ***	0.30 **	0.55 ***	-	
5. Collective action participation (Time 3)	0.11	0.12	0.24 ***	0.38 ***	-
6. Mindfulness practice experience	−0.16 **	−0.16 **	−0.12 *	−0.18 **	−0.10
Mean	4.57	4.67	4.84	3.81	2.10
SD	0.62	0.76	1.07	0.75	0.73
Cronbach’s alpha	0.83	0.88	0.90	0.78	0.92

Note: *** *p* < 0.001, ** *p* < 0.01, * *p* < 0.05.

## Data Availability

Data are available upon written requests made directly to the corresponding author.
